# Isolation and Characterization of 21 Microsatellite Loci in *Cardiocrinum giganteum* var. *yunnanense* (Liliaceae), an Important Economic Plant in China

**DOI:** 10.3390/ijms13021437

**Published:** 2012-01-31

**Authors:** Rong Li, Jie Yang, Junbo Yang, Zhiling Dao

**Affiliations:** 1 Key Laboratory of Biodiversity and Biogeography, Kunming Institute of Botany, Chinese Academy of Sciences, Kunming 650201, Yunnan, China; E-Mail: lirong@mail.kib.ac.cn; 2 Key Laboratory of Tropical Forest Ecology, Xishuangbanna Tropical Botanical Garden, Chinese Academy of Sciences, Mengla 666303, Yunnan, China; E-Mail: yangjie@xtbg.org.cn; 3 Pharmacy and Inspection Department, Sichuan Traditional Chinese Medicine College, Mianyang 621000, Sichuan, China; 4 The Germplasm Bank of Wild Species in Southwest China, Kunming Institute of Botany, Chinese Academy of Sciences, Kunming 650201, Yunnan, China

**Keywords:** *Cardiocrinum giganteum* var. *yunnanense*, microsatellite markers, polymorphism, population genetics, Liliaceae

## Abstract

Twenty-one microsatellite markers from the genome of *Cardiocrinum giganteum* var. *yunnanense*, an important economic plant in China, were developed with a fast isolation protocol by amplified fragment length polymorphism of sequences containing repeats (FIASCO). Polymorphism within each locus was assessed in 24 wild individuals from Gaoligong Mountains in western Yunnan Province, China. The number of alleles per locus ranged from 2 to 4 with a mean of 2.9. The expected and observed levels of heterozygosity ranged from 0.042 to 0.726 and from 0.000 to 1.000, with averages of 0.44 and 0.31, respectively. These polymorphic microsatellite markers should prove useful in population genetics studies and assessments of genetic variation to develop conservation and management strategies for this species.

## 1. Introduction

The herbaceous perennial genus *Cardiocrinum* (Endlicher) Lindley is a member of the lily family (Liliaceae) and contains three species and one variety, namely *Cardiocrinum cathayanum* (E. H. Wilson) Stearn, *C. cordatum* (Thunb.) Makino, *C. giganteum* (Wall.) Makino, and *C. giganteum* var. *yunnanense* (Leichtlin ex Elwes) Stearn [1]. All the species are characterized by ovate-cordate leaves with reticulate veins, and terminal racemes with many large flowers [2].

**Figure 1:**
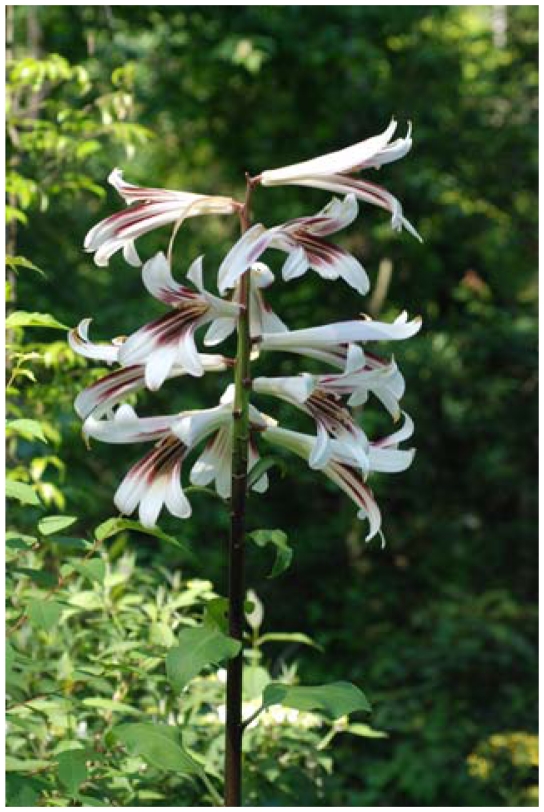
Inflorescence of *Cardiocrinum giganteum* var. *yunnanense*, showing the attractive flowers.

*Cardiocrinum giganteum* var. *yunnanense* is a perennial herb and mainly distributed in Gansu, Guangdong, Guangxi, Guizhou, Henan, Hubei, Hunan, Shaanxi, Sichuan, and Yunnan provinces of China and adjacent regions of Myanmar [3]. It grows in forests at altitudes ranging from 1200 to 3600 m elevation [3]. With its attractive flowers (Figure 1), this species has attracted the attention of botanists and horticulturalists, who have taken an interest in the commercial development of this species as an ornamental resource [4]. It is an important economic plant in China. The seeds are used as a replacement for *Aristolochia* fruits to treat cough [5], and bulbs are used as a starch staple by the local people in Guangxi and Yunnan [6]. Its economic attributes have made *Cardiocrinum giganteum* var. *yunnanense* at risk of overexploitation of natural populations. Moreover, its habitat has been badly degraded and fragmented due to heavy logging and forest destruction in past decades, which has reduced the distribution of this species to a fragmented range with small populations [7].

To provide effective conservation and management strategies for this important economic plant, it is necessary to understand the spatial genetic structure, genetic diversity, and levels of gene flow within and among its populations. However, in the genus *Cardiocrinum*, except 13 microsatellite loci were developed from the genome of *C. cordatum* [8], no nuclear microsatellite primers or other types of markers have been reported for *C. giganteum* var. *yunnanense*. Microsatellites show numerous advantages over other fingerprinting methods such as RAPD, ALFP, and ISSR because they are locus-specific, codominant, highly reproducible, and usually highly polymorphic [9]. Hence, we have developed and characterized 21 microsatellite markers for *Cardiocrinum giganteum* var. *yunnanense*, which will facilitate further investigations on the genetic diversity, population structure, and gene flow of this species.

## 2. Results and Discussion

In total, 273 positive clones were sequenced. A total of 223 (82%) sequences were found to contain simple sequence repeats (SSRs); 114 of these with appropriate microstellite and sufficient flanking regions were selected to design locus-specific primers. Polymorphisms of all 114 microsatellite loci were assessed in 24 wild individuals. Of these primers, 30 successfully amplified the target regions, and 21 of them displayed polymorphisms and 9 showed monomorphism (Table 1). The number of alleles per locus ranged from 2 to 4, with a mean of 2.9. The expected (*H*_E_) and observed (*H*_O_) heterozygosities ranged from 0.042 to 0.726 and from 0.000 to 1.000, with average of 0.44 and 0.31, respectively (Table 2). Among 21 microstellite markers, 16 loci showed significant deviation from Hardy-Weinberg equilibrium (HWE) (*P* < 0.01) (Table 2), probably due to heterozygote deficiency or the limitation of sample size, also because of the presence of null alleles. Twenty-eight loci pairwise (13.3%) showed significant genotypic linkage disequilibrium (LD) between pairs of loci (*P* < 0.001).

## 3. Experimental Section

### 3.1. Isolation of Microsatellite Loci

Genomic DNA was extracted from silica-gel-dried leaves by following a CTAB method and the microsatellite loci were isolated by using the fast isolation by AFLP of sequences containing repeats (FIASCO) protocol [10,11]. Approximately 500 ng of total genomic DNA was digested with *Mse*I enzyme (New England Biolabs, Beberly, MA, USA), and then fragments were ligated to the *Mse*I AFLP adaptor pair (5′-TACTCAGGACTCAT-3′/5′-GACGATGAGTCCTGAG-3′) at 37 °C for 2 h with T_4_ DNA ligase (Fermentas, Burlington, ON, Canada).

A diluted digestion-ligation mixture (1:10) was amplified with the adaptor-specific primers *Mse*I-N (5′-GATGAGTCCTGAGTAAN-3′) by following the program: 95 °C for 3 min, 30 cycles of 94 °C for 30 s, 53 °C for 60 s, 72 °C for 60 s followed by an elongation step of 5 min at 72 °C. Amplified fragments with a size range of 200–800 bp were enriched for microsatellite repeats by magnetic bead selection with 5′-biotinylated (AC)_15_, (AG)_15_, and (AAG)_10_ probes. Captured fragments were re-amplified with adaptor-specific primers. Polymerase chain reaction (PCR) products were purified by using an EZNA Gel Extraction Kit (Omega Bio-Tek, Guangzhou, China).

The purified PCR products with enriched microsatellite repeats were ligated into the pGEM-T vector (Promega, USA), and transformed into DH5α cells (TaKaRa, Dalian, China). Recombinant clones were screened by blue/white selection and the positive clones were tested by PCR with (AC)_10_/(AG)_10_/(AAG)_7_ and T_7_/Sp_6_ primers. The clones with positive inserts were sequenced with an ABI PRISM 3730XL DNA sequencer (Applied Biosystems, Foster City, CA, USA). The program Oligo 6.0 was used to design locus-specific primers for those microsatellite sequences found to containt sufficient flanking regions [12].

### 3.2. Detection of Polymorphism

Polymorphisms of microsatellite loci were evaluated in 24 wild individuals of *Cardiocrinum giganteum* var. *yunnanense* from Gaoligong Mountains (24°40′–28°30′ N, 98°11.2′–98°47.5′ E) in western Yunnan province. Polymerase chain reactions (PCR) were performed in 20 μL of reaction containing 30–50 ng genomic DNA, 0.6 μM of each primer, 7.5 μL 2× *Taq* PCR MasterMix [Tiangen (Tiangen, Beijing China); 0.1 U *Taq* Polymerase/μL, 0.5 mM dNTP each, 20 mM Tris-HCl (pH = 8.3), 100 mM KCl, 3 mM MgCl_2_]. PCR amplifications were conducted under the following program: 95 °C for 3 min followed by 30–36 cycles at 94 °C for 30 s, with the annealing temperature optimized for each specific primer (Table 1), for 30 s, 72 °C for 60 s, and a final extension step at 72 °C for 7 min. The amplified fragments were separated on 6% polyacrylamide denaturing gels with a 20-bp ladder molecular size standard (Fermentas, Burlington, Ontario, Canada) by silver staining.

### 3.3. Data Analysis

Standard genetic diversity parameters of polymorphic loci, e.g., the number of alleles (*N*_A_), and expected (*H*_E_) and observed levels of heterozygosity. We also estimated deviations from Hardy-Weinberg equilibrium (HWE) and genotypic linkage disequilibrium (LD) between pairs of loci using Chi-square tests.

## 4. Conclusions

The 21 microsatellite markers developed in this study are the first set of such markers for *Cardiocrinum giganteum* var. *yunnanense*. They should prove useful for further investigating the spatial genetic structure, genetic diversity, and levels of gene flow within and among populations of this species, which will help to develop viable strategies for the conservation and management of this important economic plant.

## Figures and Tables

**Table 1:** Characteristics of 30 microsatellite loci in *Cardiocrinum giganteum* var. *yunnanense*.

Locus	Primer sequences (5′-3′)	Repeat motif	Size range (bp)	*T*_a_ (°C)	GenBank Accession No.
CGY003 *	F: TATGGAGGGTTCTATTGCC	(AG)_9_	214–223	52	JQ340036
	R: GGTTTCAGAGTTCATTGGAT				
CGY005	F: AAAGGAGGGGAAGGCATAGT	(AG)_5_AT(AG)_5_	257	55	JQ340037
	R: CTAAGAGACCGCCTCCTCAT				
CGY006 *	F: TGGTATTGTCAGAATCTCAT	(TG)_10_(AG)_13_	169–182	50	JQ340038
	R: AGGTTGTTGGGTGGAGTAGT				
CGY007 *	F: TGTGTGAGTTTGAGCATAAT	(AG)_15_	130–145	52	JQ340039
	R: CGGATACTCAGTGAACCTAC				
CGY011 *	F: ACAATAACCCCAGTAGACC	(AG)_14_	164–168	55	JQ340040
	R: TGGGTGCTCATCAAGTGTC				
CGY012 *	F: CGAACTGAACATTGAGAAGA	(AG)_15_	119–127	50	JQ340041
	R: ATTACACTCTGACAACACCG				
CGY015	F: TCTCAAGTAAATCCAACAAAT	(AG)_16_	156	50	JQ340042
	R: AAGGTATTGGAATGGCGAT				
CGY028 *	F: ATGAGAAAGAGAGATGAAAG	(AG)_13_	147–153	48	JQ340043
	R: TAAAGTGTGTGTAGGTGGAG				
CGY029 *	F: TTCATTATCATCTCGGACAC	(TG)_7_(AG)_14_	157–165	48	JQ340044
	R: AGAAGGTCAACCGAACACAT				
CGY031 *	F: ACTCCTCTACCCTTTCACCA	(AG)_9_	163–173	48	JQ340045
	R: CATGATATTTATACTGAGGTTCT				
CGY035	F: AACAAAAGAAAGCAGTAGAA	(TC)_11_	184	48	JQ340046
	R: TATGATAGAGCAAAAGAGGG				
CGY036 *	F: TATCGCCTTCTTACACTTA	(AG)_18_	178–182	50	JQ340047
	R: TGAGCCGATTCCTACATTTT				
CGY037	F: TCCAAGAGAGAAAGCATCAA	(TC)_9_	144	50	JQ340048
	R: ATGGCAGAATCACAATAAGT				
CGY043 *	F: TTTCAGCCACCCTCACTATT	(AG)_8_	180–186	50	JQ340049
	R: CTCCTATTTTTACAAGACGC				
CGY053 *	F: TGCCAGAAAAGAATCACAA	(AG)_11_	144–152	50	JQ340050
	R: ATGACCCTTCCTAATTCG				
CGY054 *	F: ACCCAAATAAAGTAACAGACCA	(TC)_16_	197–207	57	JQ340051
	R: TGCCCCATCACATCCCCACC				
CGY058 *	F: GTAGTTTCCTTCATCGCCTT	(TC)_15_	243–251	57	JQ340052
	R: CCACACAGGGAGGCATCTTT				
CGY064 *	F: TATTTCTTATTCTTCACCTC	(AG)_14_	121–127	47	JQ340053
	R: AAAACCAATAAAATCCCTC				
CGY065 *	F: CCGTTGGGATTATGAGTATT	(TC)_19_	167–175	50	JQ340054
	R: CAGCATAGAGCATAGCCCTT				
CGY066 *	F: TGGAGAGATTCAGGTTCATA	(AG)_14_	218–230	52	JQ340055
	R: GAGACCATACATCACTAAATCA				
CGY067	F: GTGACCTTAGGAGTATATTAGC	(AG)_10_	237	55	JQ340056
	R: CGGAAATGGCTACTAACTAAGA				
CGY072	F: AGATGAAGGAGTAGGGACAA	(TC)_8_	305	55	JQ340057
	R: CAAACTCCCACTCACCATTC				
CGY073 *	F: GTCTCCCTCCTTCTCAAAAT	(TC)_7_	250–258	55	JQ340058
	R: CTTCTTGCCCCCACTAACTT				
CGY075 *	F: GCCATAGAGACATAGGGAGG	(AG)_19_	213–221	55	JQ340059
	R: ATGAAACCTGACCTAAGCAC				
CGY083 *	F: CCTACTCATTTTTCAACTTTC	(TG)_13_	290–298	52	JQ340060
	R: GCCCATTCCCAACCACTATT				
CGY091 *	F: TGGACACATTTTTGGCTAAG	(AC)_5_AT(AC)_11_	120–132	50	JQ340061
	R: CGACGATTAGGGCAAAGGTA				
CGY099	F: TCATTCCACTCCACCATAAA	(AC)_12_	119	50	JQ340062
	R: ATACCTAACCATCTTCCAAT				
CGY105	F: CCCAAAAATAATCATCAAGC	(AC)_7_	152	52	JQ340063
	R: CACCTACCCTGCTTTGTTCA				
CGY110 *	F: ATAGTGTAGCAGTGAAGCGA	(TG)_7_	115–119	57	JQ340064
	R: TGTGGTTGGTTTCTCATTGC				
CGY111	F: TGACACCCCCATACTTAGAC	(TG)_7_	115	50	JQ340065
	R: TCTCATCACTCTATCTCATT				

*T*_a_: PCR annealing temperature;

* displayed polymorphisms.

**Table 2:** Result of 21 polymorphic microsatellite loci screening in 24 wild individuals of *Cardiocrinum giganteum* var. *yunnanense*.

Locus	*N*_A_	*H*_E_	*H*_O_	*F*_is_		Locus	*N*_A_	*H*_E_	*H*_O_	*F*_is_	
	
				W&C	R&H					W&C	R&H
CGY003 *	2	0.042	0.042	–	–	CGY054 *	2	0.042	0.042	–	–
CGY006 *	3	0.434	0.042	0.906	0.493	CGY058 *	3	0.543	0.917	−0.715	−0.411
CGY007	4	0.611	0.875	−0.446	−0.243	CGY064 *	2	0.550	0.083	0.851	0.486
CGY011 *	4	0.621	0.292	0.536	0.370	CGY065 *	3	0.657	0.125	0.813	0.882
CGY012 *	4	0.726	0.458	0.374	0.439	CGY066 *	2	0.156	0	1.000	1.044
CGY028	4	0.482	0.292	0.401	0.357	CGY073 *	2	0.337	0	1.000	1.044
CGY029 *	3	0.635	0.083	0.871	0.928	CGY075 *	3	0.511	0	1.000	1.044
CGY031 *	2	0.350	0.083	0.766	0.847	CGY083 *	3	0.624	1	−0.624	−0.489
CGY036	3	0.254	0.125	0.514	0.531	CGY091 *	2	0.042	0.042	–	–
CGY043	3	0.669	0.583	0.131	0.155	CGY110	3	0.465	0.417	0.107	−0.068
CGY053 *	3	0.566	1	−0.795	−0.489						

*N*_A_: number of alleles; *H*_E_: expected heterozygosity; *H*_O_: observed heterozygosity; *F*_is_: estimates of inbreeding coefficient; W&C: Weir and Cockerham’s method; R&H: Robertson and Hill’s method;

* statistically significant deviation from Hardy-Weinberg equilibrium (HWE) (*P* < 0.01).
